# Therapeutic Study of Cinnamic Acid Derivative for Oxidative Stress Ablation: The Computational and Experimental Answers

**DOI:** 10.3390/molecules28217425

**Published:** 2023-11-04

**Authors:** Oluwafemi Adeleke Ojo, Akingbolabo Daniel Ogunlakin, Rotdelmwa Filibis Maimako, Gideon Ampoma Gyebi, Christopher Busayo Olowosoke, Odunayo Anthonia Taiwo, Tobiloba Christiana Elebiyo, David Adeniyi, Bolaji David, Matthew Iyobhebhe, Juliana Bunmi Adetunji, Damilare IyinKristi Ayokunle, Adebola Busola Ojo, Ramzi A. Mothana, Abdullah R. Alanzi

**Affiliations:** 1 Good Health and Wellbeing Research Cluster, Bowen University, Iwo 232102, Nigeria; gbolaogunlakin@gmail.com (A.D.O.); pelumidavid676@gmail.com (D.A.); tkdavid29@gmail.com (B.D.); 2 Phytomedicine, Molecular Toxicology, and Computational Biochemistry Research Laboratory (PMTCB-RL), Department of Biochemistry, Bowen University, Iwo 232101, Nigeria; 3 Department of Biochemistry, Landmark University, Omu-Aran 251101, Nigeria; maimako.rotdelmwa@lmu.edu.ng (R.F.M.); elebiyotobiloba@gmail.com (T.C.E.); iyobhebhematthew@gmail.com (M.I.); 4 Natural Products and Structural (Bio-Chem)-Informatics Research Laboratory (NpsBC-RI), Department of Biochemistry, Bingham University, Karu 961105, Nigeria; gideonagyebi@gmail.com; 5 Department of Biotechnology, Federal University of Technology, PMB 704 Futa Road, Akure 340252, Nigeria; olowosokechris@gmail.com; 6 Department of Biotechnology, Chrisland University, Abeokuta 110118, Nigeria; 7 Department of Biochemistry, Chrisland University, Abeokuta 110118, Nigeria; odunayotaiwo25@gmail.com; 8 Department of Biochemistry, Osun State University, Osogbo 232106, Nigeria; adetunjibj@gmail.com; 9 Department of Pure and Applied Biology, Bowen University, Iwo 232101, Nigeria; opeoluwa02@gmail.com; 10 Department of Biochemistry, Ekiti State University, Ado-Ekiti 362103, Nigeria; adebolaojo04@gmail.com; 11 Department of Pharmacognosy, College of Pharmacy, King Saud University, P.O. Box 2457, Riyadh 11451, Saudi Arabia; rmothana@ksu.edu.sa (R.A.M.); aralonazi@ksu.edu.sa (A.R.A.)

**Keywords:** cinnamic acid, oxidative stress, liver, purinergic function, molecular docking simulation, density functional theory, ADMET profiling

## Abstract

This study aimed to examine the therapeutic activity of the cinnamic acid derivative KAD-7 (N′-(2,4-dichlorobenzylidene)-3-(4-methoxyphenyl) acrylohydrazide) on Fe^2+^-induced oxidative hepatic injury via experimental and computational models. In addition, the role of ATPase and ectonucleoside triphosphate diphosphohydrolase (ENTPDase) in the coordination of cellular signals is speculated upon to proffer suitable therapeutics for metabolic stress disorder upon their inhibition. While we know little about therapeutics with flexible dual inhibitors for these protein targets, this study was designed to screen KAD-7’s (N′-(2,4-dichlorobenzylidene)-3-(4-methoxyphenyl) acrylohydrazide) inhibitory potential for both protein targets. We induced oxidative hepatic damage via the incubation of hepatic tissue supernatant with 0.1 mM FeSO_4_ for 30 min at 37 °C. We achieved the treatment by incubating the hepatic tissues with KAD-7 under the same conditions. The catalase (CAT), glutathione (GSH), malondialdehyde (MDA), ATPase, and ENTPDase activity were all measured in the tissues. We predicted how the drug candidate would work against ATPase and ENTPDase targets using molecular methods. When hepatic injury was induced, there was a significant decrease in the levels of the GSH, CAT, and ENTPDase (*p* < 0.05) activities. In contrast, we found a noticeable rise in the MDA levels and ATPase activity. KAD-7 therapy resulted in lower levels of these activities overall (*p* < 0.05), as compared to the control levels. We found the compound to have a strong affinity for ATPase (−7.1 kcal/mol) and ENTPDase (−7.4 kcal/mol), and a better chemical reactivity than quercetin. It also met all drug-likeness parameters. Our study shows that KAD-7 can protect the liver from damage caused by FeSO_4_ by reducing oxidative stress and purinergic actions. Our studies indicate that KAD-7 could be developed as a therapeutic option since it can flexibly inhibit both ATPase and ENTPDase.

## 1. Introduction

Plants contain a vast array of phytochemicals that are not just essential to them but also beneficial to man. These phytochemicals are frequently produced as defenses against harsh environmental conditions, prey, pests, and diseases [1]. These phytochemicals are plant secondary metabolites that validate the use of plants as natural therapies [2].

Polyphenols are an important group of phytochemicals that comprise stilbenes, coumarins, tannins, flavonoids, phenolic acids, and lignans. Phenolic acids represent benzoic and cinnamic acid derivatives [3].

Cinnamic acid is an organic acid that is primarily found in the bark of the cinnamon tree. Recent research has shown that the acid and all its derivatives possess physiological effects that include protection of the nervous and cardiovascular systems, and their antioxidant, anticancer, antiviral, antidiabetic, and antifungal properties [3,4]. Among its other pharmacological properties, cinnamic acid and its derivatives are reported to ameliorate diabetes and its complications as they are reported to exhibit hypoglycemic and antioxidant activities. The mechanism of its antidiabetic action includes gluconeogenesis inhibition, stimulation of insulin secretion, stimulation of glucose absorption, and delay of carbohydrate digestion, among others [3,5]. These compounds demonstrate their antioxidant activity by preventing the cleavage of hydrogen ions, which results in the neutralization of free radicals [3,5]. Ogunlakin et al. [5] reported the effect of KAD-7, a derivative of cinnamic acid, on the proliferation of the CHO-1 and HeLa cell lines (with abnormal *p53* tumour suppressor gene). The backbone structure of KAD-7 (Figures S1 and S2), which has hydrazones, has been the focus of substantial investigation in recent years due to its numerous features and possible uses in the areas of pharmaceutical, chemical, and analytical chemistry [6]. Due to their numerous biological characteristics, such as antibacterial [7,8,9], antifungal [10], and anticancer [11,12,13,14], hydrazone compounds are becoming more and more common.

Oxidative stress is caused by the huge production of reactive oxygen species (ROS), also called free radicals. Excess free radicals have the potential to harm biomolecules such as DNA, proteins, and lipids [15,16]. Antioxidants are, on the other hand, compounds that prevent the action of free radicals. They function by acting directly to inactivate the free radicals or by the reduction of oxidized molecules. A considerable imbalance between free radicals and the antioxidant scavenging system is considered a significant factor in the pathogenesis and progression of a number of diseases and aging [16].

The effect of the liver on the body’s metabolic processes is well known. As the liver is the principal organ necessary for maintaining and controlling homeostasis, as such, it plays a crucial role in the excretion of endogenous and exogenous chemicals as well as the metabolism of carbohydrates, proteins, and fats. It is also a key player in maintaining iron homeostasis as iron is an essential cofactor and is involved in most metabolic processes [17]. Iron carries out several of its functions via its unique ability to assume the ferric (Fe^3+^) and ferrous state (Fe^2+^). This dual functional state also makes iron essential in the cell’s redox homeostasis. Impaired iron regulation leading to excessive hepatic iron storage further elevates the production of free radicals. This elevated production results in oxidative stress, which ultimately leads to oxidative hepatotoxicity. Numerous liver disorders, including cirrhosis, hepatitis, hepatocellular carcinoma, and fibrosis, have been related to oxidative injury as a major mechanism of etiology and progression [18].

This study aims to investigate the therapeutic effect of the cinnamic acid derivative, KAD-7, for oxidative stress ablation via an experimental and computational approach.

## 2. Results

### 2.1. Antioxidant Activity

**Figure 1:**
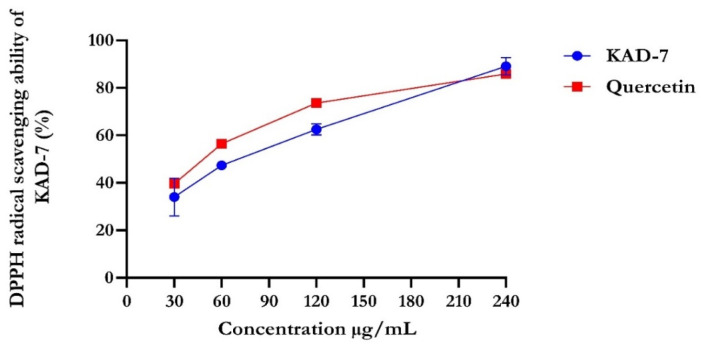
DPPH scavenging ability of N′-(2,4-dichlorobenzylidene)-3-(4-methoxyphenyl) acrylohydrazide. Data expressed as mean ± SD (n = 3) Legends: KAD-7: N′-(2,4-dichlorobenzylidene)-3-(4-methoxyphenyl) acrylohydrazide.

**Figure 2:**
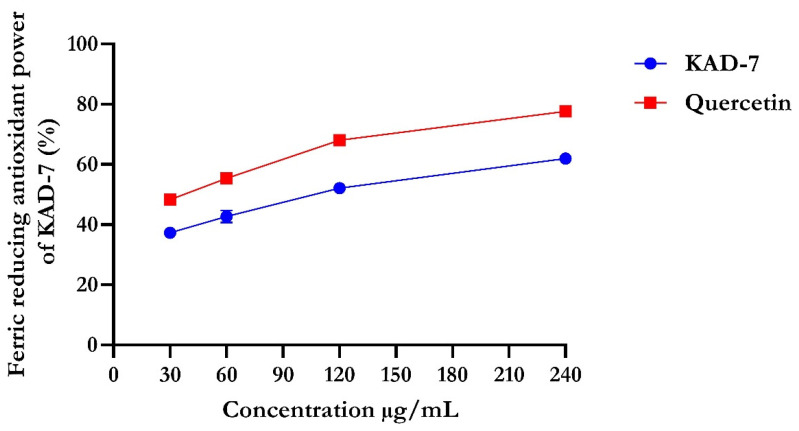
Ferric-reducing antioxidant power of N′-(2,4-dichlorobenzylidene)-3-(4-methoxyphenyl) acrylohydrazide. Data expressed as mean ± SD (n = 3). Legends: KAD-7: N′-(2,4-dichlorobenzylidene)-3-(4-methoxyphenyl) acrylohydrazide.

**Figure 3:**
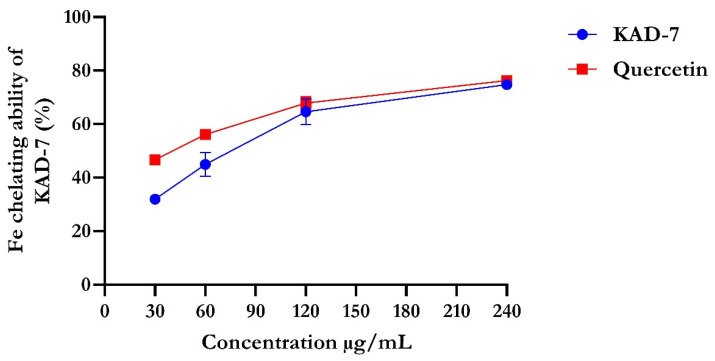
Iron-chelating ability of N′-(2,4-dichlorobenzylidene)-3-(4-methoxyphenyl) acrylohydrazide. Data expressed as mean ± SD (n = 3). Legends: KAD-7: N′-(2,4-dichlorobenzylidene)-3-(4-methoxyphenyl) acrylohydrazide.

The percentage of DPPH radical scavenging activity of KAD-7 compared favorably with quercetin, as seen in Figure 1. The scavenging activity showed an increase with increasing concentrations of KAD-7. The ferric-reducing antioxidant power of KAD-7 also compared favorably with that of the standard (quercetin) (Figure 2). Furthermore, the antioxidant activity increased in a dose-dependent manner. KAD-7 demonstrated a high iron-chelating ability that increased dose dependently and compared favorably to the standard (quercetin) (Figure 3).

### 2.2. Ex Vivo Antioxidant Activity

**Figure 4:**
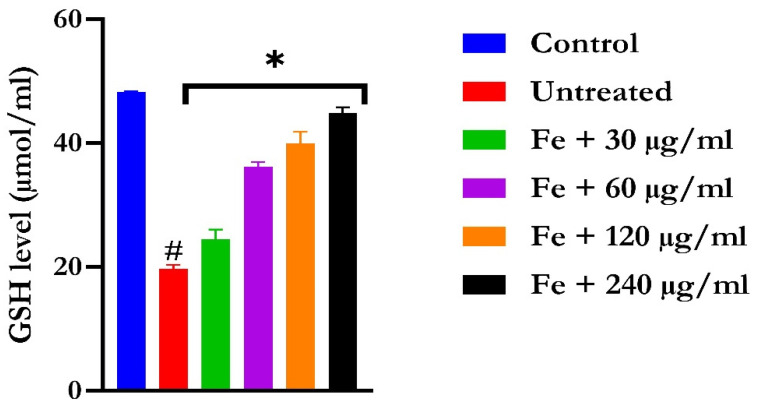
The impact of N′-(2,4-dichlorobenzylidene)-3-(4-methoxyphenyl) acrylohydrazide on the glutathione (GSH) levels in FeSO_4_-induced liver toxicity. Data = mean ± SD; n = 3. * Signifies significant difference in comparison with the untreated tissue; # signifies significant difference in comparison with the normal tissue.

**Figure 5:**
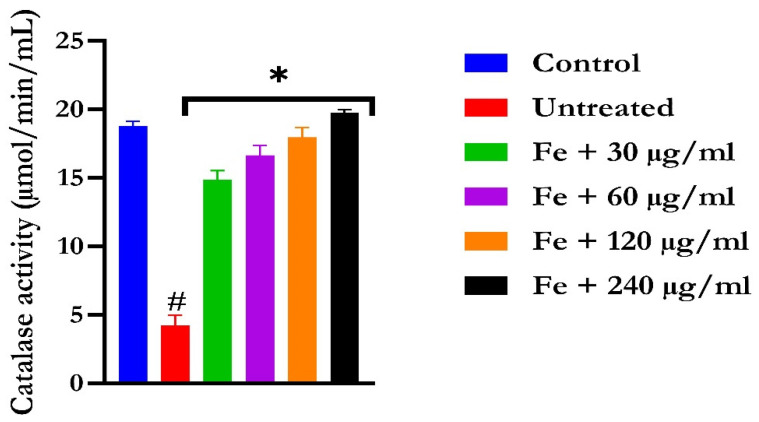
The impact of N′-(2,4-dichlorobenzylidene)-3-(4-methoxyphenyl) acrylohydrazide on catalase activity in FeSO_4_-induced liver toxicity. Data = mean ± SD; n = 3. * Signifies significant difference in comparison with the untreated tissue; # signifies significant difference in comparison with the normal tissue.

**Figure 6:**
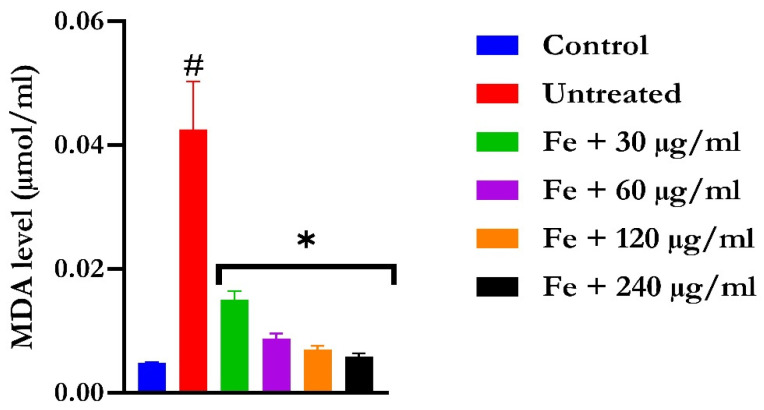
The impact of N′-(2,4-dichlorobenzylidene)-3-(4-methoxyphenyl) acrylohydrazide on MDA level in FeSO_4_-induced liver toxicity. Data = mean ± SD; n = 3. * Signifies significant difference in comparison with the untreated tissue; # signifies significant difference in comparison with the normal tissue.

The induction of oxidative hepatic injury resulted in a significant (*p* < 0.05) decrease in the GSH level and catalase activity and a concomitant increase in MDA levels, as shown in Figure 4, Figure 5, and Figure 6, respectively. Treatment with KAD-7 compared favorably with the control and reversed these parameters to near normal in a dose-dependent manner.

### 2.3. Purinergic Function

**Figure 7:**
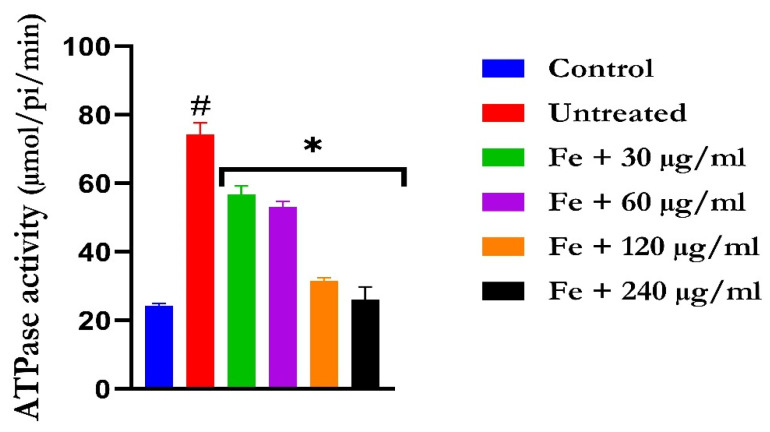
The impact of N′-(2,4-dichlorobenzylidene)-3-(4-methoxyphenyl) acrylohydrazide on ATPase activity in FeSO_4_-induced liver toxicity. Data = mean ± SD; n = 3. * Signifies significant difference in comparison with the untreated tissue; # signifies significant difference in comparison with the normal tissue.

**Figure 8:**
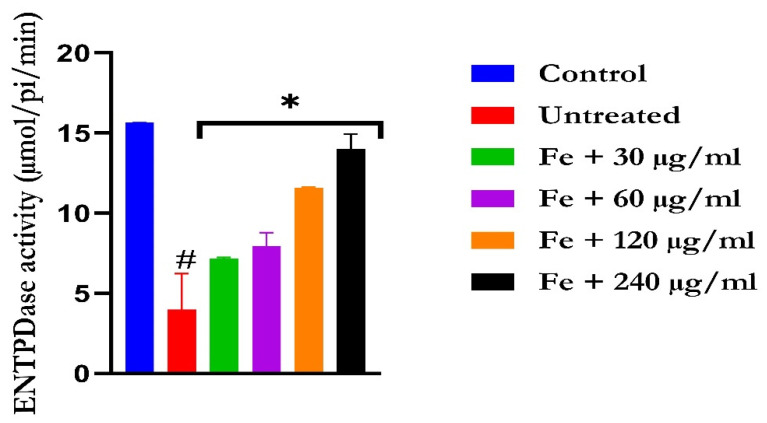
The impact of N′-(2,4-dichlorobenzylidene)-3-(4-methoxyphenyl) acrylohydrazide on ENDTPase activity in FeSO_4_-induced liver toxicity. Data = mean ± SD; n = 3. * Signifies significant difference in comparison with the untreated tissue; # signifies significant difference in comparison with the normal tissue.

The induction of oxidative hepatic injury led to a significant (*p* < 0.05) increase in the hepatic ATPase activity with a concomitant decrease in the ENTPDase activity. Treatment with KAD-7 led to a significant reversal of both activities to near normal in a dose-dependent manner and compared favorably with the control (quercetin), as depicted in Figure 7 and Figure 8, respectively.

### 2.4. ADME/Toxicity Profiling of Ligands

**Figure 9:**
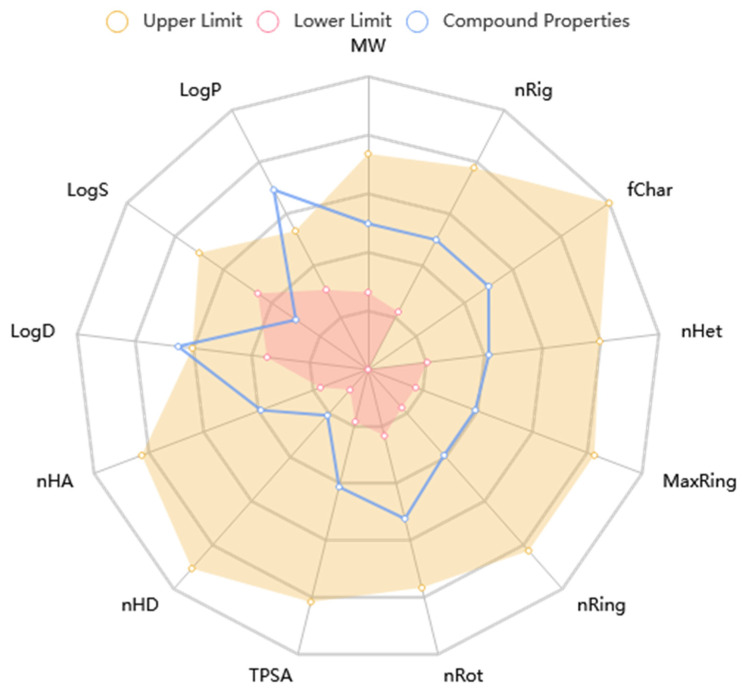
The radar plot of in silico physicochemical KAD-7. The radar plot demonstrates the physicochemical properties of KAD-7 (in blue) and the reference optimal scope (in red and yellow).

The KAD-7 N′-(2,4-dichlorobenzylidene)-3-(4-methoxyphenyl) acrylohydrazide) prediction showed that there was excellent absorption and carbonate permeability, leading to improved bioavailability ratings. The compounds were able to pass across the blood–brain barrier, had a huge capacity of distribution (VD), had low plasma protein binding (less than 92 percent), and did not act as an inhibitor or substrate for glycoprotein (Table 1). It is anticipated that the excretion characteristics of the molecule will consist of a 0.112 half-life, as well as a low clearance rate (4.116). Based on the acute oral toxicity potential, the chemical has an insufficiently unfavorable toxicity profile for toxin generation, and it was further classed as stage III (somewhat toxic) based on this potential, with the exception of hepatotoxicity and nephrotoxicity. The Lipinski, Ghose, Veber, and Egan guidelines were not broken in the making of this chemical; nonetheless, it does have the potential to be used as a drug. Nonetheless, the violation of the XLOGP3>3.5 property indicates the candidate as having a poor lead-likeness (Table 1). Figure 9 shows that KAD-7 demonstrated physicochemical properties that were within the reference optimal range.

### 2.5. 2D/3D Interaction

**Figure 10:**
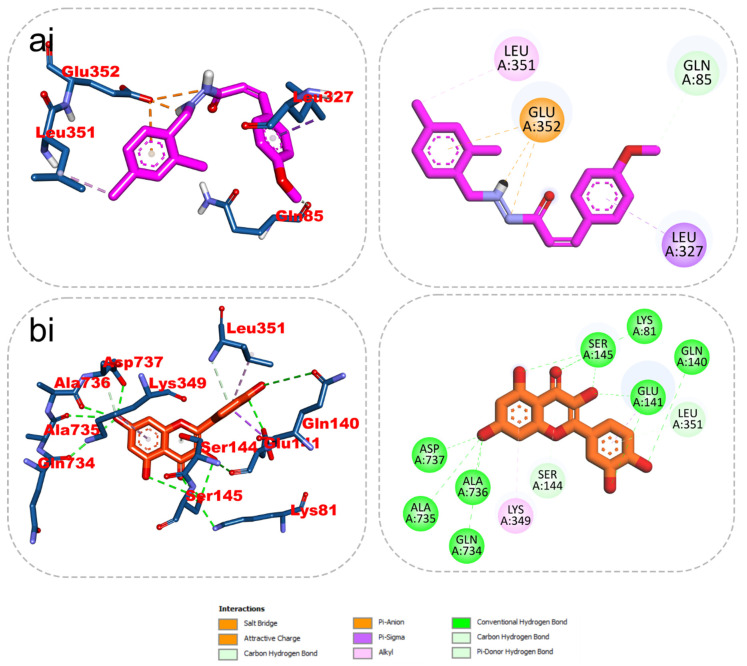
The 2D/3D interactions of reference compound (quercetin) and KAD-7 in the binding site of ATPase. Stick representations of the ligands are shown by colors (**ai**) (upper) purple: KAD-7 and (**bi**) (lower) gold: quercetin.

**Figure 11:**
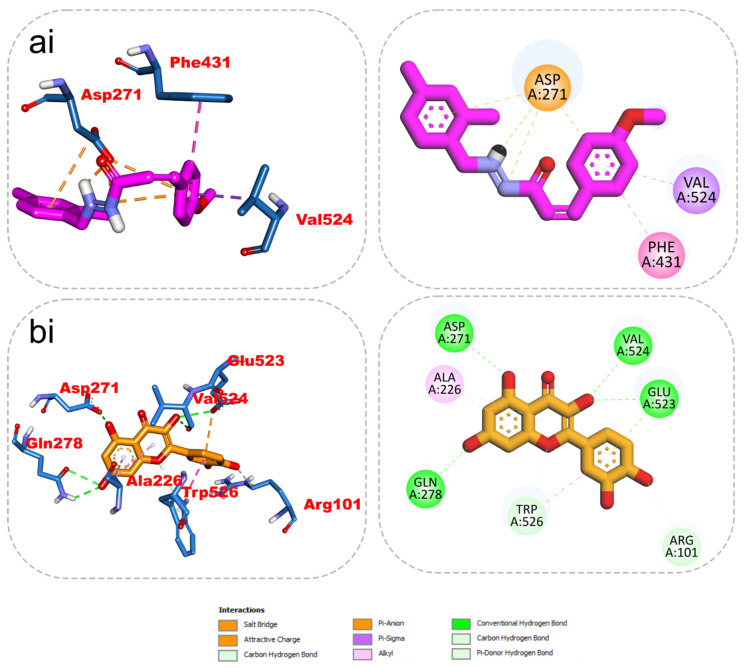
The 2D/3D interactions of reference compound (quercetin) and KAD-7 in the binding site of ENTPDase. Stick representations of the ligands are shown by colors (**ai**) (upper) purple: KAD-7 and (**bi**) (lower) gold: quercetin.

The results from the docking analysis of KAD-7 and the reference compounds are presented in Table 2. It was observed that KAD-7 had a close binding energy to the quercetin, the reference compound. The amino acid interaction of the ligands (KAD-7 and quercetin) with the amino acid residues of the target proteins is presented in the same table. The pictorial representation of both the 3D and 2D interaction is further depicted in Figure 10 and Figure 11. Altogether, the test ligand interacted with similar amino acids as the reference compounds, showing that they were docked into the same binding site of the proteins. 

### 2.6. Molecular Dynamics Simulation

**Figure 12:**
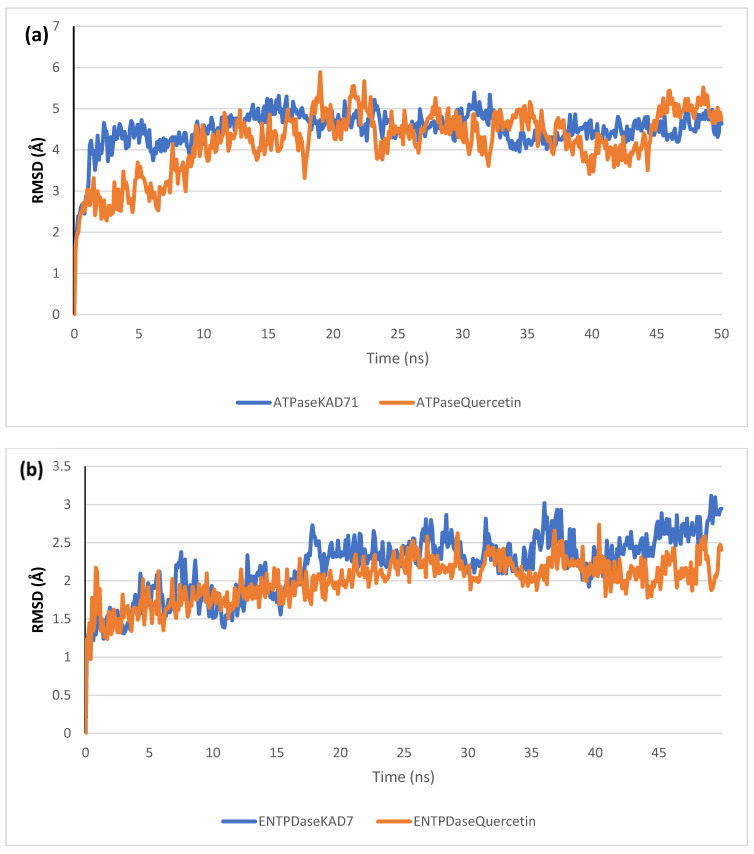
The backbone root-mean-square deviation (RMSD) plots of molecular dynamics (MD) simulation of ligands complexed to (**a**) ATPase and (**b**) ENTPDase.

**Figure 13:**
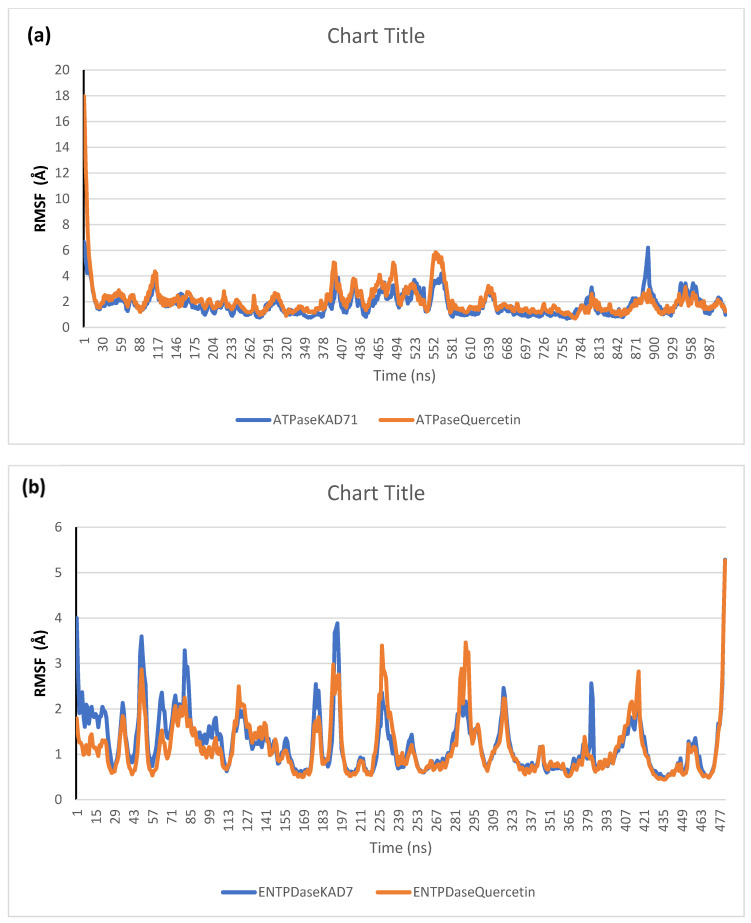
Per residue root-mean-square fluctuation (RMSF) plots of molecular dynamics (MD) simulation of ligands complexed to (**a**) ATPase and (**b**) ENTPDase.

**Figure 14:**
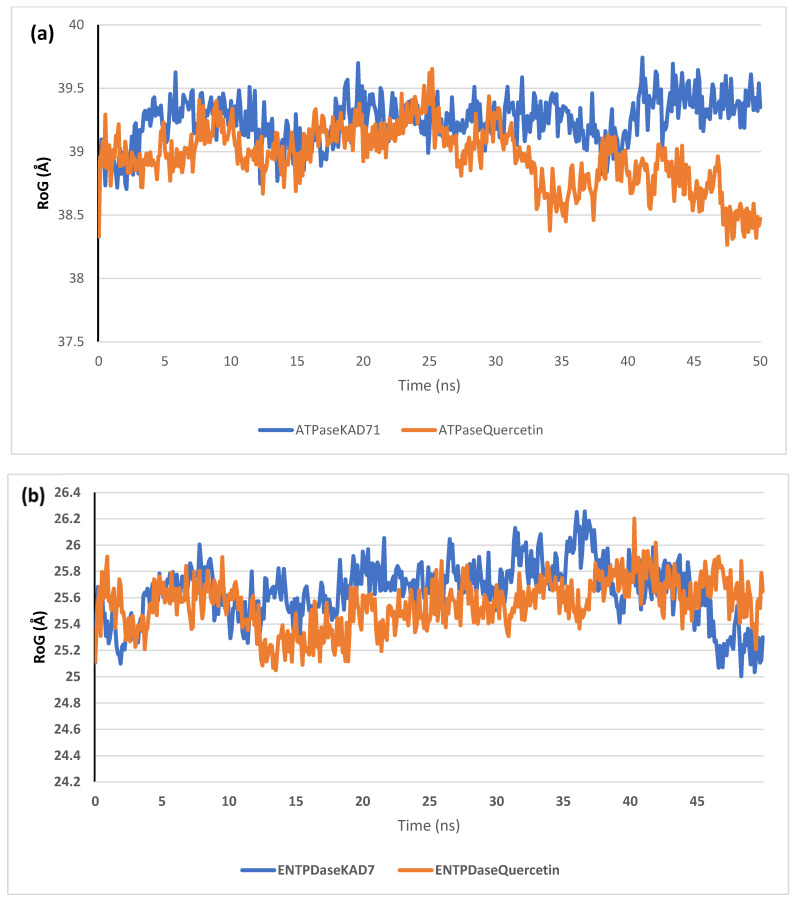
The radius of gyration (RoG) plots of molecular dynamics (MD) simulation of ligands complexed to (**a**) ATPase and (**b**) ENTPDase.

**Figure 15:**
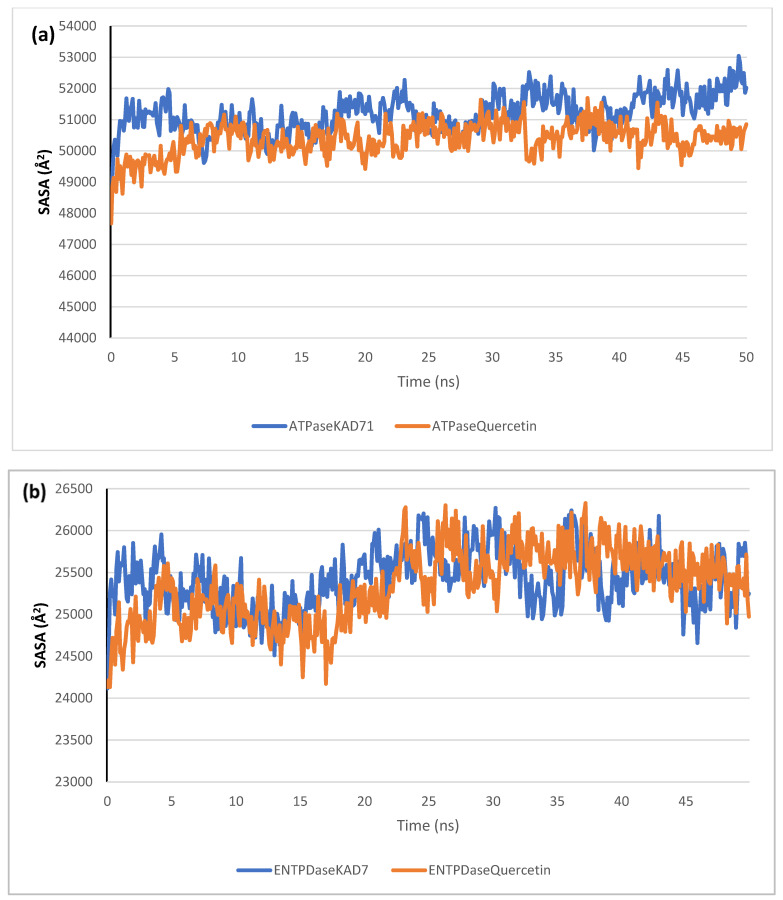
The solvent-accessible surface area (SASA) plots of molecular dynamics (MD) simulation of ligands complexed to (**a**) ATPase and (**b**) ENTPDase.

**Figure 16:**
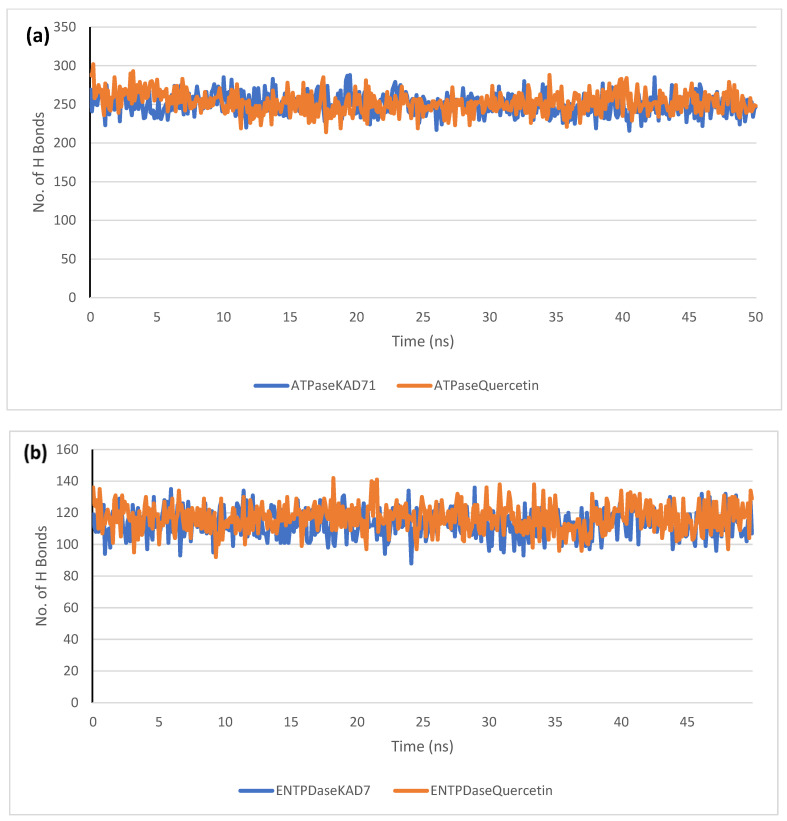
The changes in the number of H-bonds during the MDS trajectory of ligands complexed to (**a**) ATPase and (**b**) ENTPDase.

The stability of the complexes formed between KAD-7 and the two proteins were compared with those formed with the reference compound in a full atomistic MDS. The trajectory obtained from the MDS were further analyzed using the Tk console scripts in VMD version 1.9.3. The RMSD, RMSF, RoG, SASA, and number of H-bonds were analyzed. The computed means of each of the parameters alongside the standard deviations are presented in Table 3. Also, the spectra plot for the complex systems are presented in Figure 12, Figure 13, Figure 14, Figure 15 and Figure 16. The RMSD plots for the ATPase and ENTPDase complexes were equilibrated before 10 ns, after which, the simulation progressed with a minimal fluctuation (of less than 3 Å) until the end of the simulation (Figure 12). The KAD-7- and quercetin-bound systems presented a similar progression of the RMSF spectra. The ligand-bound systems also presented close mean RMSF values. (Figure 13). The RoG plots show that the ATPase and ENTPDase complexes were equilibrated before 10 ns with a minimal fluctuation throughout the period of the simulation (Figure 14). The KAD-7- and quercetin-bound systems presented very close mean RoG values. The SASA plots of the complexes of the ATPase and ENTPDase complexes demonstrated a minimal fluctuation during the period of simulation. This was further corroborated by the very close mean SASA values (Figure 15). The number of H-bonds during the simulation was fairly constant with minimal fluctuations. The ligand-bound complexes presented a close number of hydrogen bonds (Figure 16).

## 3. Discussion

Antioxidants are essential health-enhancing chemicals that combat the actions of free radicals (reactive oxygen and nitrogen species). The strong antioxidant activity of KAD-7 can be inferred from its DPPH, FRAP, and Fe-chelating antioxidant properties [19], which indicate that it could be used as a rich source of natural antioxidants.

Hepatic oxidative injury in excised liver tissue was achieved via incubation with FeSO_4_ in the presence of KAD-7 and quercetin. The decrease in the GSH level and catalase activity is indicative of pro-inflammation, indicating the induction of oxidative injury. This is further evidenced by the elevated hepatic MDA level, which is indicative of peroxidation of the hepatic lipids following the induction of oxidative hepatic injury. These altered levels and activity are in sync with the report of [18] that showed the occurrence of oxidative imbalance after the induction of oxidative hepatic injury in an isolated liver. The Fenton and Haber–Weiss reactions, which are catalyzed by iron, triggered the onset of oxidative imbalance as a result of the incubation of the hepatic tissue with FeSO_4_ [18]. Treatment with KAD-7 confirmed its beneficial role in hepatic oxidative injury as evidenced by decreased MDA levels and increased GSH and SOD activity, implying an anti-peroxidative and antioxidative effect.

An imbalance in the purinergic enzyme activity is indicative of hepatic injury. The phospho-hydrolysis of adenosine monophosphate (AMP) and adenosine triphosphate (ATP) is catalyzed by the purinergic enzymes in order to release the endogenous signaling nucleotide (adenosine), which is reported to be a key factor in bioenergetics [18,20]. In this present study, the elevated ATPase activity due to the induction of oxidative hepatotoxicity is indicative of a depleted hepatic ATP activity, while the depletion in ENTPDase activity suggests a depletion in adenosine levels [20]. The treatment with KAD-7 caused a reversal of these activities, thus indicative of the compound’s modulatory activity on purinergic functions in oxidative hepatotoxicity. Previous studies have also reported the hepato-protective role of cinnamic acid and its derivatives [21].

The transmembrane protein sodium potassium pump (Na^+^/K^+^ ATPase) is an ion-pumping complex that regulates the osmotic balance function. Also, it acts as a scaffold in complex eukaryote cells [22]. Similarly, ecto-nucleoside triphosphate diphosphohydrolases (E-NTPDases), a family of enzymes found on the cell surface and lumen of certain organelles, regulate purinergic signaling and perform certain protein synthesis [23,24]. While studies have linked both enzymes to the mediation of oxidative stress agents (ROS) related to human pathologies like obesity, atherosclerosis, heart failure, uremic cardiomyopathy, hypertension, and cancer [24,25,26], a novelty approach has been proven with their inhibition, thus bridging the signaling pathway associated with these diseases [27,28]. Their inhibitory study is currently being planned with a major therapeutic intervention [24,29].

From this study, the evaluation of the physicochemical and ADME/Tox properties of a precursor for drug design and development was carried out on the synthesized cinnamic derivative [30]. The compound excretion properties are predicted to have a short half-life and a low clearance rate. The compound exhibits a drug-likeness potential, with neither of the Lipinski, Ghose, Veber, and Egan rules violated. Nonetheless, the violation of the XLOGP3>3.5 property indicates the candidate as having a poor lead-likeness.

The molecular docking shows how KAD-7 N′-(2,4-dichlorobenzylidene)-3-(4-methoxyphenyl) acrylohydrazide forms an interaction with the ATPase and ENTPDase targets. Collectively, the in silico screening demonstrated that our derivative has considerable pharmacological properties responsible for the reactive oxidative specie-scavenging attribute. As a result, it might be taken into account for the development of an alternative therapy for abating oxidative stress.

Computer-aided drug development technologies, such as molecular docking, have recently been used mostly for the early screening of prospective therapeutic candidates derived from phytochemical medicinal plants [31]. The interaction and orientation of ligands in the binding site of known receptors are predicted using this tool. [32]. The result from the docking analysis shows that KAD-7 has the tendencies of strongly interacting with the binding site of both enzymes. The residues involved in these binding interactions have been highlighted in our previous studies to strongly correlate with the inhibitory tendencies that were demonstrated against both enzymes in the in vitro studies. Furthermore, the stability of bound complexes is important to the overall interaction within the complex, hence the need for stability analysis. 

The RMSD plots reveal the extent of the deviation of each frame from the initial structure, hence they are used to access the protein stability of the systems. The results from the RMSD plots show that the complexes formed with KAD-7 were stabile during the course of the simulation. This further indicates that the integrity of the complexes was preserved [33]. The RMSF plots reveal the flexibility of different regions of the enzymes, The binding of KAD-7 did not alter the internal flexibility of the proteins [34]. The compactness of the bound systems was further measured from the analysis of the RoG plots. Usually, the SASA plots show how much solvent is accessible from the protein’s surface. In order to determine if the integrity of the folded protein is affected by the binding of ligands, RoG and SAS are both used [35]. Both analyses showed that the compactness of the protein structures was not compromised. 

Additionally, the results from the predictive pharmacokinetic and ADMET studies showed that KAD-7 fulfilled all the requirements for all the filtering tools that were used to access the pharmacokinetic potential. The compound’s ability to pass all the requirements for the five filtering tools shows that the compound is highly druggable [36,37,38]. KAD-7 established good absorption properties, with a high probable intestinal absorption [39]. Substances that inhibit the hERG channel may have negative effects on the heart because it is well known that the hERG channel is essential for the repolarization and termination stages of the action potential in cardiac cells. The lack of a hERG channel blocker potential in KAD-7 suggests that they could not trigger hERG channel-related cardiotoxicity [40,41]. KAD-7 also has good distribution potential [42]. The BBB is described as a barrier protecting the brain through a “physical” and “biochemical” barrier made up of enzyme activity that serves as a crucial barrier between the systemic circulation and the central nervous system (CNS) [36]. One of the main problems with the administration of CNS medications has been noted by [43]. KAD-7 tested positively for its capacity to penetrate the blood–brain barrier (BBB), one of the several molecular properties that were investigated in silico, adding to the evidence that it can reach the brain, where its neuroprotective impact is most required. This study investigated the impact of KAD-7 on drug biotransformation using multiple cytochrome P450 descriptors [40]. KAD-7 was predicted to be in the range of classified LD_50_ values [44,45] and did not exhibit carcinogenicity [46]. 

## 4. Materials and Methods

### 4.1. Chemicals

KAD-7 (N′-(2,4-dichlorobenzylidene)-3-(4-methoxyphenyl) acrylohydrazide), synthesized from cinnamic acid by Ogunlakin [5], was used in this study. Quercetin was made by SantaCruz Biotechnology, which is based in Heidelberg, Germany. All of the other chemicals were good for lab work.

### 4.2. Antioxidant Studies

#### 4.2.1. DPPH Radical Scavenging Activity of KAD-7

DPPH assay of KAD-7 was assessed following the procedure illustrated by [47]. In this experiment, 0.5 mL of varying concentrations of KAD-7 was mixed with 1 mL of freshly prepared 0.2 mM DPPH solution in absolute methanol. The mixture was incubated at room temperature for 30 min before the absorbance was read at 517 nm using a UV–vis spectrophotometer. Quercetin was used as the standard in this study. The percentage inhibition was calculated as follows:

$$Percentage\;inhibition(\%)=1-\left[\frac{Abs\;of\;sample}{Abs\;of\;control}\right]$$

#### 4.2.2. Ferric-Reducing Antioxidant Power of KAD-7

FRAP assay of KAD-7 was determined by the procedure illustrated by [47] with slight modifications. In brief, 250 µL of varying concentrations of the plant sample was added to 0.625 mL of 0.2M phosphate buffer (pH 6.6) and 0.625 µL of 1% K_3_Fe(CN)_6_. Following incubation at 500 °C for 20 min and subsequent cooling of the reaction mixture at room temperature, 0.625 mL of 10% trichloroacetic acid was added to halt the reaction. Afterward, the mixture was centrifuged at 2000× *g* for 10 min; 0.625 mL of the supernatant was pipetted into a clean test tube containing 0.625 mL of distilled water and 125 µL of 1% FeCl_3_. The mixture was allowed to stay for 10 min and the absorbance was read at 700 nm against a blank. 

#### 4.2.3. Evaluation of Iron (Fe)-Chelating Activity of KAD-7

Iron (Fe)-chelating assay of KAD-7 was determined using the methods described by [48] with slight modifications. Approximately 500 µL of 0.2 mM FeCl_3_ was added to 100 µL varying concentrations of KAD-7 and standard (EDTA). The reaction was activated by adding 200 µL of 5 mM ferrozine to the mixture, which was subsequently incubated at 25 °C for 10 min. The absorbance was measured at 562 nm.

### 4.3. Experimental Rats and Organ Preparation

The Department of Anatomy at Bowen University provided healthy male Wistar rats weighing between 250 and 300 g for this study. The university is located in Nigeria. After the rats had fasted for the previous night, they were put to death with halothane, and their livers were removed before being combined with 1% Triton X-100 in a solution containing 50 mM phosphate. The homogenate was spun in a centrifuge at 15,000 revolutions per minute at a temperature of 40 degrees Celsius. The rats were cared for in accordance with the guidelines established by the Institutional Animal Ethics Committee of Bowen University. This study was approved by the committee under the approval number BUI/BCH/2022/001. This ensured that the ethical treatment and care of the animals were maintained throughout this study. 

### 4.4. Induction of Liver Damage

The procedures described in [49] were used, with only a few alterations here and there, to bring about liver damage in an in vitro model. In a nutshell, 200 L of the organ supernatant with different concentrations of KAD-7 (ranging from 30–240 g/mL) was mixed with 100 L of 0.1 mM FeSO_4_ to form the final mixture. The samples were then put through a series of biochemical tests after an incubation period of thirty minutes at 37 °C. Positive control involved tissue supernatant and FeSO_4_, while negative control used organ supernatant in reaction mixtures.

### 4.5. Antioxidant Activities

#### 4.5.1. Catalase (CAT) Activity of KAD-7

CAT activity assay of KAD-7 was evaluated following the description of [49] with slight modifications. 

#### 4.5.2. Reduced Glutathione Level

A total of 600 µL of the tissue lysates was deproteinized using 10% trichloroacetic acid as outlined in [50]. The mixture was then subjected to centrifugation at a speed of 3500 rpm for a duration of 10 min. After this centrifugation step, 500 µL of the resulting sample was carefully transferred into a clean test tube. Subsequently, 100 µL of the Ellman reagent was added to the test tube containing the sample. The absorbance of the solution was read at 415 nM. 

#### 4.5.3. Lipid Peroxidation Level

This study aimed to assess the potential of KAD-7 to reduce lipid peroxidation using a method described in [49]. In this methodology, a reaction mixture was prepared and subjected to boiling for a duration of 60 min at a temperature of 95 °C. Subsequently, the mixture was allowed to cool down and the absorbance of the solution was read at a wavelength of 532 nanometers (nm). 

### 4.6. Purinergic Activity

The ATPase and ENTPDase activities of KAD-7 were determined using the methods described in [49,51].

### 4.7. In Silico Studies

#### 4.7.1. 3D Structure of Protein and Determination of Binding Pocket

The human unique ID for the Alphafold model was found in the Uniprot database. The X-ray crystallographic structures of the ATPase (P13637) and ENTPDase (Q9Y227) enzymes were sourced from the Uniprot database. To determine the locations of unknown binding pockets in both proteins, two online tools were utilized. The first tool employed was the FTsite server, accessible at https://ftsite.bu.edu (accessed on 30 September 2023). The second tool was the Protein Plus server, available at https://proteins.plus/#dogsite (accessed on 30 September 2023). The FTsite and Protein Plus server are two experimentally validated webservers designed on the algorithm of comparison of standard HETATM available on protein banks similar to our query protein and scoring with the best appropriate catalytic sites presented with a suitable PyMol-generated session to visualize the binding pocket. By using these tools, this study aimed to identify and characterize the binding pockets in the ATPase and ENTPDase enzymes [52,53]. With the help of the PyMol programming package, the amino acid residues were extracted from the PSE session file of the projected result.

#### 4.7.2. Retrieval of N′-(2,4-Dichlorobenzylidene)-3-(4-methoxyphenyl) acrylohydrazide) and Quercetin

KAD-7 N′-acrylohydrazide and quercetin were sourced from their respective databases.

#### 4.7.3. ADME/Toxicity Profiling of Ligands

Pharmacokinetic behavior refers to the processes of absorption, distribution, metabolism, excretion, and toxicity associated with a pharmaceutical compound [54]. The ADME/toxicity profiles of ligands were determined following the procedure described by [30].

#### 4.7.4. Molecular Docking Simulation

The molecular docking simulation analysis was carried out as previously reported [55,56]. Before the simulation analysis, the ligands were prepared using USCF chimera to add the polar hydrogen and charge. Using the python prescription software (https://pyrx.sourceforge.io/) for the analysis, the ENTPdase Grid center: x; 5.6189, y; 9.5081, z; 9.2914; Grid Dimension: 28.6533; y; 25.0000; z; 39. 27.4334 and ATPase (Alphafold ID; AF-P13637-F1) Grid center: x; 5.1462, y; −5.1996, z; −10.6907; Grid Dimension: 27.1597; y; 32.8993; z; 39. 1517 were generated for both active protein sites initially determined using FTsite server. 

#### 4.7.5. Molecular Dynamics Simulation

The stability of complexes formed between the test ligands (KAD-7 and quercetin) and the target proteins (ATPase and ENDtase) was studied in a full 50 ns atomistic molecular dynamics. The preferred time (ns) was selected because of the size of the ATPase protein with more than 1000 amino acid residues. The same protocol as published in our recent publication was adopted for this study [57,58]. Using the CHARMM36m force field, the parameters for amino acids in proteins, water molecules, and ions were determined. On the other hand, the small molecules were parameterized using the CHARMM general force field (CGenFF) tool included in the CHARMM-GUI [59,60,61]. GROMACS 2020.3 was employed for the simulation [62]. The systems were solvated in a cubic box using the TIP3P water model with a 1 nm padding, and then neutralized by adding NaCl ions at a concentration of 0.154 M [62] was utilized as an MD package to perform the simulation with periodic boundary conditions (PBCs) applied in the three directions. Initially, the steepest descent technique was used to minimize the system’s potential energy and eliminate atomic collisions with the maximal allowed force set to 100 KJ·mol^−1^·nm^−1^ and the number of steps set to 100,000 minimization steps. Starting with an NVT ensemble (where the number of atoms, volume, and temperature are all held constant), we equilibrated the temperature to 310 K using the V-rescale method [63]. While the pressure was set to 1 atmospheric pressure and was maintained through Berendsen barostat. Finally, a production run of 100 ns in NVT ensemble was performed. In each step, the bond lengths of hydrogen-bonded atoms was constrained using the LINear Constraint Solver (LINCS) algorithm [64]. The calculation of electrostatics was performed using the Particle Mesh Ewald (PME) (8) algorithm with a cutoff of 1.2 nm. The Newtonian equations of motion were integrated using a leap-frog method, with a time step of 1 femtosecond for the equilibration stages and 2 femtoseconds for the production steps. Every 0.1 ns of the production run, a frame was recorded, summing to 1000 frames per system. The trjconv command was used to remove the PBC before the trajectory was examined. TCL scripts were used with the VMD TK terminal to examine the production run [65]. The radius of gyration (RoG), solvent-accessible surface area (SASA), number of hydrogen bonds, separation of center of mass of ligand and protein, root-mean-square deviation (RMSD) for the protein alone, the ligand alone, and the protein–ligand complex were all calculated. 

### 4.8. Statistical Analysis

The descriptive statistics were shown using the SD (standard deviation) of mean (Mean ± SD). In addition, a one-way ANOVA with Tukey’s post hoc analysis and a significance threshold of *p* < 0.05 was performed. Graphpad prism version 9.0.1 was used to analyze the data.

## 5. Conclusions

Finally, these investigations show that KAD-7 may be employed for several purposes in treating liver damage caused by oxidative stress. KAD-7’s therapeutic potential in liver damage prevention stems from oxidative stress regulation. Furthermore, the current study’s molecular docking, ADME/Tox, and DFT analyses indicate that KAD-7 has a flexible inhibitory posture against ATPase and entonucleoside triphosphate diphosphohydrolase (ENTPDase) targets that is favorable for drug design.

## Figures and Tables

**Table 1:** Adme/tox properties of quercetin derivatives.

Compounds/ ADMET-Profile	Properties	KAD-7 N′-(2,4-Dichlorobenzylidene)-3-(4-methoxyphenyl)acrylohydrazide N′-(2,4-Dichlorobenzylidene)-3-(4-methoxyphenyl)acrylohydrazide)
Physicochemical	Formula	C17H14Cl2N2O2
	Molecular weight (g/mol)	349.21
	H-bond acceptors	3
	H-bond donors	1
	Rotatable bond	6
	TPSA (Å^2^)	50.69
	AlogP	4.17
	Water solubility (LogS)	−4.325
Absorption	XLOGP3	4.48
	WLOGP	4.06
	SILICOS-IT	4.87
	Human intestinal absorption	+0.9927
	Caco_2_	+0.6696
	Human oral bioavailability	+0.5857
	P-gp inhibitor	−0.6796
	P-gp substrate	−0.8773
Distribution	Volume distribution (L/Kg)	0.668
	Plasma protein binding (100%)	93.8
	Blood–brain barrier	+0.9804
Metabolism	OATP2B1 inhibitor	−0.8521
	OATP1B1 inhibitor	+0.9248
	OATP1B3 inhibitor	+0.9501
	MATE1 inhibitor	−0.9400
	OCT2 inhibitor	−0.6000
	BSEP inhibitor	+0.9097
	CYP3A4 substrate	+0.5923
	CYP2C9 substrate	−0.6150
	CYP2D6 substrate	−0.8428
	CYP3A4 inhibition	+0.5625
	CYP2C9 inhibition	+0.8115
	CYP2C19 inhibition	+0.9337
	CYP2D6 inhibition	−0.8913
	CYP1A2 inhibition	+0.9318
Excretion	Clearance	4.116
	Half-life	0.112
Toxicity	Carcinogenicity (binary)	−0.6718
	Hepatotoxicity	+0.7250
	Respiratory toxicity	−0.6333
	Reproductive toxicity	−0.5778
	Mitochondrial toxicity	−0.6750
	Nephrotoxicity	+0.4681
	Acute oral toxicity (c)	III 0.7012
	Ames mutagenesis	−0.5200
Drug-likeness	Lipinski violation	Nil
	Ghose violation	Nil
	Veber violation	Nil
	Egan violation	Nil
Medicinal Chemistry	PAIN violation	0
	BRENK violation	2
	Lead-likeness	No (XLOGP3 > 3.5)

**Table 2:** Binding affinity of the protein–ligand.

Compounds	Protein	Hydrogen Bonds Bond Distance (Å)		**Hydrophobic Interaction**
		Binding Energies	Interacted Residues	Interacted Residues
KAD-7	*ATPase*	−7.1		Leu351, Glu352, Gln85, Leu327
Quercetin		−7.9	Asp737, Ala735, Ala736, Gln734, Ser145, Lys81, Glu141, Gln140	Lys349, Glu141
KAD-7	*ENTPDase*	−7.4		Asp271, Phe431, Val524
Quercetin		−7.8	Asp271, Val524, Glu523, Gln278	Ala226, Trp526, Glu523

**Table 3:** The mean and standard deviation of different parameters analyzed from the MDS trajectories of top docked compounds complexed with respective targets.

	RMSD	RMSF	RoG	SASA	H-Bonds
	Mean (Å)	Mean (Å)	Mean (Å)	Mean (Å)	Mean (Å)
ATPase_KAD-7	4.48 ± 0.18	1.80 ± 0.83	39.24 ± 0.18	51,187.90 ± 598.62	250.07 ± 13.04
ATPase_Quercetin	4.19 ± 0.31	2.09 ± 1.26	38.95 ± 0.24	50,378.98 ± 514.39	252.57 ± 13.68
ENTPDase_KAD-7	2.19 ± 0.64	1.23 ± 0.64	25.64 ± 0.22	25,420.13 ± 347.37	113.45 ± 8.23
ENTPDase_Quercetin	2.00 ± 0.29	1.14 ± 0.61	25.54 ± 0.18	25,341.01 ± 432.89	116.78 ± 8.51

## Data Availability

Data available on reasonable request.

## Supplementary Materials

The following supporting information can be downloaded at: https://www.mdpi.com/article/10.3390/molecules28217425/s1. Figure S1: Mass spectroscopy of KAD-7; Figure S2: lH NMR of KAD-7.
