# Supplementary material for: Therapeutic Study of Cinnamic Acid Derivative for Oxidative Stress Ablation: The Computational and Experimental Answers
Source: Molecules. 2023 Nov 4;28(21):7425. doi: 10.3390/molecules28217425 (PMC10648207; doi:10.3390/molecules28217425)
Supplement: Supplementary file 1 [file molecules-28-07425-s001.zip › molecules-2681453-supplementary.pdf]

# Therapeutic study of cinnamic acid derivative for oxidative stress ablation: The computational and experimental answers

**Oluwafemi Adeleke Ojo [1,2*], Akingbolabo Daniel Ogunlakin [1,2], Rotdelmwa Filibis Maimako [3], Gideon Ampoma Gyebi[4], Christopher Busayo Olowosoke [5,6], Odunayo Anthonia Taiwo [7], Tobiloba Christiana Elebiyo [3], David Adeniyi [1,2], Bolaji David [1,2], Matthew Iyobhebhe [3], Juliana Bunmi Adetunji[8], Damilare IyinKristi Ayokunle [9], Adebola Busola Ojo [10], Ramzi A. Mothana[11] Abdullah R. Alanzi [11]**

[1] Bowen University SDG 03 (Good Health and Wellbeing Research Cluster), Nigeria.
[2] Phytomedicine, Molecular Toxicology, and Computational Biochemistry Research Laboratory (PMTCB-RL), Department of Biochemistry, Bowen University, Iwo, 232101, Nigeria
[3] Department of Biochemistry, Landmark University, Omu-Aran, Nigeria; RFM: maimako.rotdelmwa@lmu.edu.ng; MI: iyobhebhematthew@gmail.com
4 Natural products and Structural (Bio-Chem)-Informatics Research Laboratory (NpsBC-RI), Department of Biochemistry, Bingham University, Karu *961105*, Nigeria; gideonagyebi@gmail.com
[5] Department of Biotechnology, Federal University of Technology, Akure. PMB 704 Futa Road, Ondo, Nigeria, CBO: olowosokechris@gmail.com
[6] Department of Biotechnology, Chrisland University, Abeokuta, Nigeria
[7] Department of Biochemistry, Chrisland University, Abeokuta, Nigeria; odunayotaiwo25@gmail.com
[8] Department of Biochemistry, Osun State University, Osogbo, Nigeria; JBA: adetunjibj@gmail.com
[9] Department of Pure and Applied Biology, Bowen University, Iwo, Nigeria, DIA: opeoluwa02@gmail.com
[10] Department of Biochemistry, Ekiti State University, Ado-Ekiti, Nigeria; ABO: adebolaojo04@gmail.com
[11] Department of Pharmacognosy, College of Pharmacy, King Saud University, P.OBox 2457, Riyadh 11451, Saudi Arabia.
* Correspondence: oluwafemiadeleke08@egmail.com; Tel.: +2347037824647

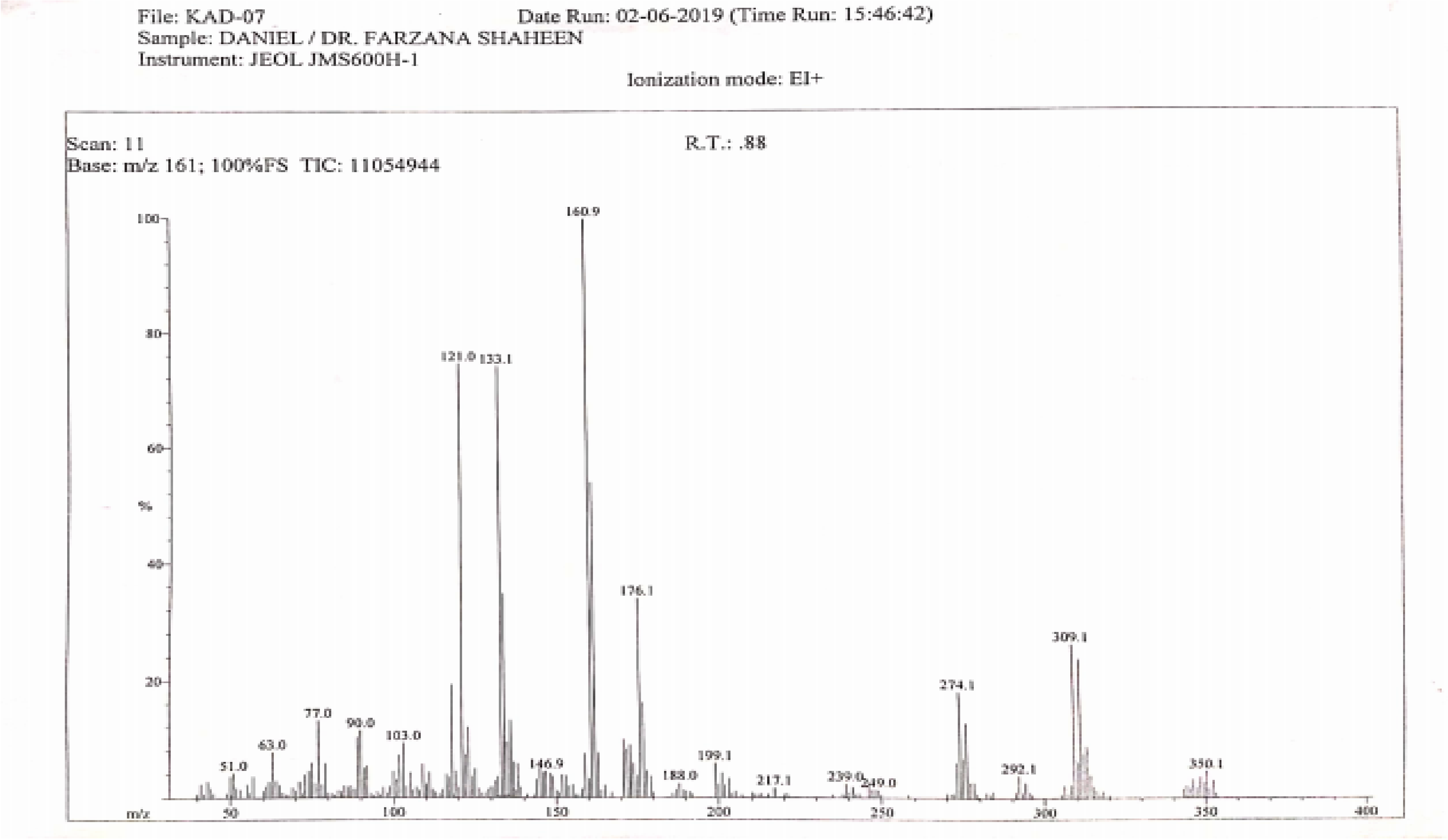


Figure S1: Mass spectroscopy of KAD-7

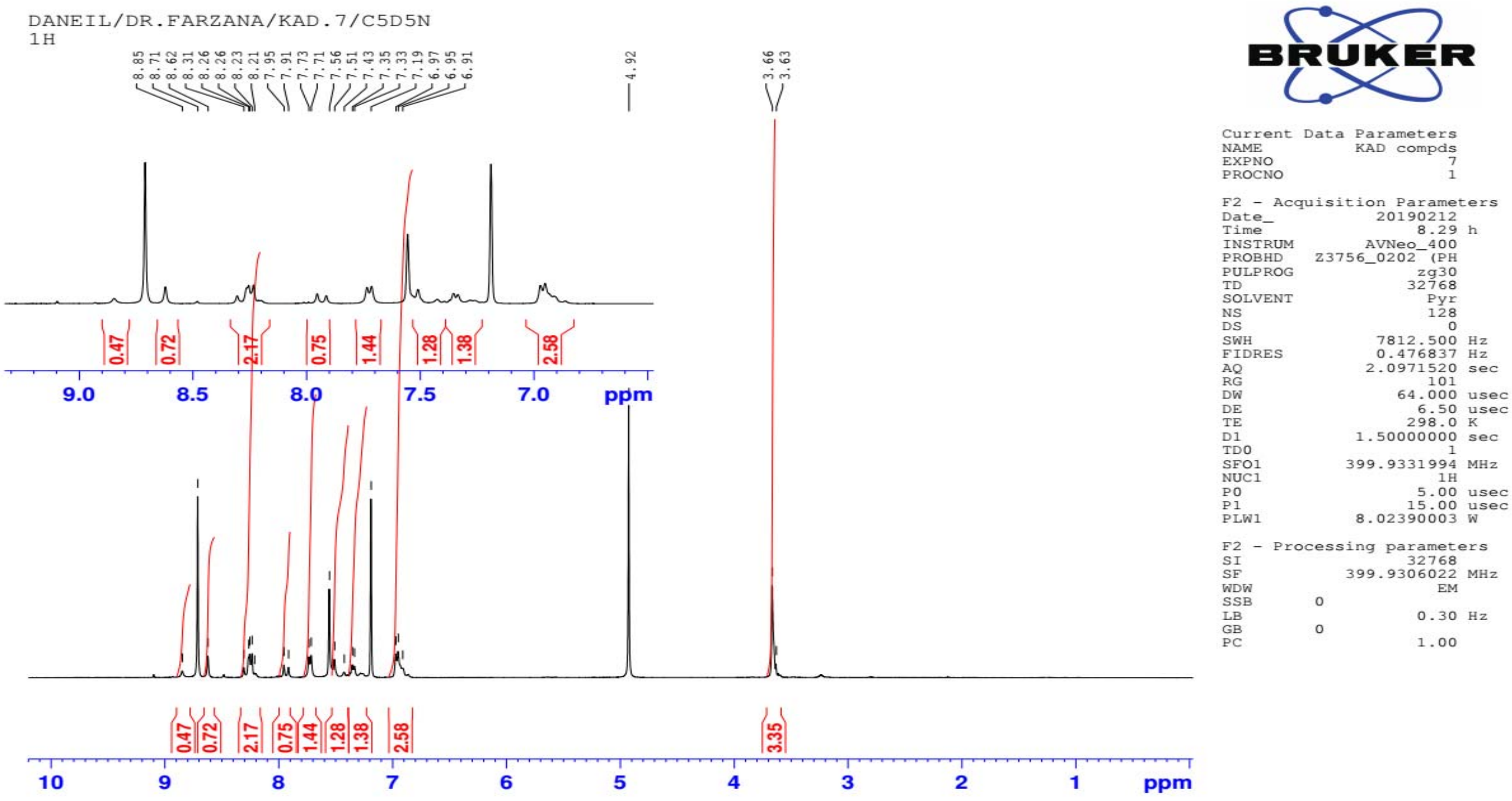


Figure S2: $^{1}$H NMR of KAD-7
